# Improvement of *Cordyline terminalis* micropropagation and bioactive compounds using biochar associated with pain and inflammation relief

**DOI:** 10.3389/fphar.2026.1882659

**Published:** 2026-08-14

**Authors:** Fadia El Sherif, Norah Ahmed Aldhafeeri, Mohamed S. Khattab, Maged E. Mohamed, Salah Khattab, Nancy Safwat Younis

**Affiliations:** 1 Department of Biological Sciences, King Faisal University, Al-Ahsa, Saudi Arabia; 2 Fab Lab, Abdulmonem Al Rashed Humanitarian Foundation, Al-Ahsa, Saudi Arabia; 3 Department of Clinical Pharmacy, Suez Canal University, Ismailia, Egypt; 4 Department of Pharmaceutical Sciences, College of Clinical Pharmacy, King Faisal University, Al-Ahsa, Saudi Arabia

**Keywords:** cordyline, *in vitro* propagation, inflammation, network pharmacology, pain, palm waste biochar

## Abstract

**Introduction:**

*Cordyline terminalis* is an important ornamental and medicinal plant valued for its antioxidant, antimicrobial, anti-inflammatory, antiproliferative, hypolipidemic, and wound-healing activities outcomes. Biochar is a carbonaceous substance derived from the controlled pyrolysis of biomass with limited oxygen availability and is commonly used in plant tissue culture to improve nutrient availability and adsorb toxic compounds. This study evaluates the effect of biochar supplementation, applied individually or in combination with 6-benzylaminopurine (BAP), on *in vitro* proliferation of *C. terminalis* Another goal of the existing study is to explore the targets of *C. terminalis* in the amelioration of pain and inflammation.

**Material and Methods:**

Randomized design with six treatments was employed in this experiment to estimate the effect of biochar on *in vitro* multiplication of *C. terminalis*. Shoot tips were cultured on MS media supplemented with 0, 4, or 6 g/L biochar, with or without 1.0 mg/L BAP. Growth responses including shoot, leaf and root number, plant height, explant fresh weight and root length were assessed. Air dried plantlets from each treatment were processed to obtain methanolic extracts for GC–MS analysis to evaluate the effect of biochar on phytochemical composition. Network pharmacology approach was conducted to recognize the promising molecular targets of *C. terminalis* and to clarify their mechanistic association with pain and inflammation.

**Results:**

Biochar enhanced *in vitro* growth, physiological performance, and secondary metabolite accumulation, supporting its potential for sustainable plant propagation and metabolite production. Explants cultured with 1 mg/L BAP combined with 4 g/L biochar showed the highest shoot proliferation, leaf number, and explant fresh weight. In contrast, biochar alone (4–6 g/L) enhanced shoot elongation, root development, and increased chlorophyll and carotenoid contents. GC–MS analysis indicated that 6 g/L biochar with BAP improved the diversity and abundance of bioactive compounds, including glucosides and sterol-related metabolites. Venn diagram included 414 common targets which are the potential therapeutic targets through which *C. terminalis* may exert anti-inflammatory and analgesic effects. PPI enrichment suggested that the action of *C. terminalis* against inflammation/pain is likely multi-target and multi-pathway. Gene Ontology enrichment analysis of the common targets supported multi-target and multi-pathway mechanism, in which the studied bioactive compounds may modulate inflammation and pain by regulating transcription factors, cytokine signaling, kinase activity, nuclear receptor pathways, and cellular stress responses.

## 1 Introduction

*Cordyline terminalis* is a widely grown ornamental plant known for its attractive and colorful leaves, making it suitable for both indoor decoration and outdoor landscaping. It belongs to the family Agavaceae and is commonly cultivated in tropical and subtropical regions as a potted plant, landscape species, and cut foliage crop (Khan et al., 2004; Jena et al., 2025). *C. terminalis* (syn. *Cordyline fruticosa*) is commonly known as the ti plant, Hawaiian ti plant, good luck plant, or cabbage palm in different regions and horticultural practices (Tematio Fouedjou et al., 2023a).

Currently, plants are valuable sources of anti-inflammatory agents as they provide structurally various, biologically active, and frequently multi-target compounds that can modify inflammatory pathways at several levels. *C. terminalis* flower is traditionally used to treat asthma (Bogoriani et al., 2022) and as a swelling medicine in Bali (Adaku et al., 2020). Several studies showed that *C. terminalis* and its metabolites exhibited anti-inflammatory. For instance, leaf extract of *C. terminalis* exhibited potent activity against carrageenan induced Wistar rat edema (Noor et al., 2022). Similarly, *C. terminalis* flower is used to treat asthma, known allergic inflammatory condition of the airways (Adaku et al., 2020).

Several studies showed that *C. terminalis* and it is metabolites exhibited analgesic activity. For instance, this plant is commonly used by certain populations as a decoction to relieve pain caused by benign prostatic hyperplasia (José et al., 2025). In addition to its ornamental importance, several species of Cordyline are known for their medicinal and biological properties due to the presence of bioactive compounds such as flavonoids, steroidal saponins, glycosides, phenolics, and sapogenins. These phytochemicals have been reported to exhibit antioxidant, antimicrobial, anti-inflammatory, antiproliferative, hypolipidemic, and wound-healing activities (Tematio Fouedjou et al., 2023a; Bogoriani et al., 2021; Phuong Ng et al., 2021).

Traditional propagation methods of *C. terminalis* such as seeds and stem cuttings are limited because they produce non-uniform plants and require longer periods for multiplication, making them less efficient for large-scale commercial production (Jena et al., 2025). Plant tissue culture is an effective method for the rapid propagation of genetically uniform, disease-free plantlets of *C. terminalis* throughout the year. It also supports the conservation and mass production of elite ornamental genotypes with desirable growth and leaf color characteristics. Various regeneration methods, including axillary bud proliferation, organogenesis, and somatic embryogenesis, have been established using different explants and culture conditions; however, protocol standardization remains challenging due to genotype and explant variability (Sudheer et al., 2022; Ray et al., 2012).

Biochar has recently gained attention as a promising additive in plant tissue culture due to its porous structure, high surface area, and rich carbon content (Di Lonardo et al., 2013; Nazeer et al., 2025). It is a stable black carbonaceous material produced through the pyrolysis of biomass under oxygen-limited conditions using agricultural residues, forestry waste, and organic by products (Varkolu et al., 2025). Owing to its unique physicochemical properties, biochar has been widely utilized in agriculture, environmental remediation, water treatment, carbon sequestration, and renewable energy production (Afshar and Mofatteh, 2024; Khan et al., 2024). In agriculture, biochar improves soil fertility, nutrient availability, water retention, and reduces environmental stress, contributing to sustainable crop production and climate change mitigation (Ogunwa et al., 2026).

In plant tissue culture, biochar can enhance nutrient absorption, improve medium aeration and moisture retention, adsorb toxic metabolites, and reduce phenolic-induced tissue browning (Mustika et al., 2025). It has also been reported to promote shoot proliferation, root induction, chlorophyll synthesis, and overall plant growth while serving as a low-cost and eco-friendly alternative to activated charcoal in micropropagation systems (Di Lonardo et al., 2013; Wiszniewska et al., 2023a). There is a documented association between inflammation and pain in different disease such as knee osteoarthritis (Dainese et al., 2022), Fibromyalgia (García-Domínguez, 2025) and Endometriosis (Cuffaro et al., 2024) among other diseases. By understanding the role of biochar in enhancing nutrient availability and improving the microenvironment within the culture medium, this study seeks to determine its potential as a sustainable additive to optimize micropropagation efficiency. Particular attention is given to its effect on increasing the multiplication rate. The study also focuses on promoting healthier and more vigorous shoot development, thereby supporting environmentally friendly and time efficient tissue culture practices.

Network pharmacology defines disease mechanisms as networks best targeted by multiple, synergistic drugs. The use of network pharmacology is crucial in understanding the mechanism of action of herbal medicines. Network pharmacology is evolving as a Frontier in drug discovery and development as it integrates systematic medicine with information science (Noor et al., 2022). The present study investigates the potential of biochar as an ecofriendly additive for optimizing the micropropagation of *C. terminalis*. The study evaluates its effects on shoot multiplication, shoot vigor, plantlet quality, and the accumulation of bioactive compounds. Additionally, a network pharmacology approach is employed to elucidate the molecular targets and mechanisms underlying the analgesic and anti-inflammatory activities of *C. terminalis*, providing insights into its therapeutic potential while supporting sustainable tissue culture practices.

## 2 Materials and methods

### 2.1 Response of *C. terminalis* cultures to palm waste biochar during growth and multiplication stages

Shoot tip measuring 0.5–1 cm in height, derived from two-month-old *in vitro* cultured explants, were obtained from the tissue culture laboratory at the Department of Biotechnology, Faculty of Agriculture, King Faisal University, were sub-cultured into 200 mL culture vessels, each containing 30 mL of Murashige and Skoog (MS) medium supplemented with 3% (w/v) sucrose and 6 g/L agar. Five different media treatments were prepared a control (MS medium with 1 mg/L benzyladenine [BA] (Hassan and Abdallah, 2015), MS medium was supplemented with palm waste biochar (Gwen Company, Saudi Arabia), whose physicochemical characteristics (Table 1) were provided by the manufacturer based on standard analytical quality reports. Treatments consisted of biochar at 4 or 6 g/L, as well as MS medium supplemented with 1 mg/L BA in combination with biochar at 4 or 6 g/L. The culture vessels were maintained in a growth chamber at 24 °C ± 2 °C under a 16-h photoperiod with a light intensity of 4000 lux (Philips TLM 40 W/33RS). Each treatment consisted of 10 vessels, with two shoot tips per vessel. After 4 weeks of incubation, various growth parameters were recorded, including explant fresh weight, the height of the longest shoot, and the number of shoots, leaves, and roots per explant, as well as root length. Additionally, some explants from each treatment group were air-dried at room temperature for methanol extraction to analyze phytochemical content using gas chromatography-mass spectrometry (GC/MS).

**TABLE 1:** Characteristics of palm waste biochar.

Physicochemical characteristic	Percentage (%)
Nitrogen	3.18
Phosphorus	2.49
Potassium	4.7
C:N	24.1
Carbone	46
Organic matter	50.47
pH	7.47–7.26

### 2.2 Photosynthetic pigments determination

Chlorophyll a (Chl-a), chlorophyll b (Chl-b), and carotenoid contents in leaf samples were determined colorimetrically following the method described by (A.O.A.C., 1984). Leaf samples were collected from three randomly selected explants per treatment.

### 2.3 GC/MS analysis

GC/MS analysis was carried out at the Department of Chemistry, College of Science, King Faisal University. Air-dried shoots of *C. terminalis* were extracted using 96% methanol according to the method described previously (Alphianti et al., 2025a). 100 mg of the obtained extracts were reconstituted in hexane, filtered, analyzed using a GC/MS-QP 2010 Plus instrument (Shimadzu, Japan) equipped with an RTX®-5SilMS column (30 m × 0.25 mm × 0.1 μm). Compound identification was based mass fragmentation patterns in comparison with Wiley 330.000 and NIST08 spectral libraries. The relative percentage of each detected compound was estimated from the corresponding GC peak area following the procedure of Lee et al., (Lee et al., 2018), with minor modifications.

### 2.4 Network pharmacology

Firstly, the pharmacokinetic properties, drug-likeness, and physicochemical characteristics of each compound of *C. terminalis* were reclaimed from the Swiss ADME database. Compounds were selected for target prediction based on Full compliance with Lipinski’s Rule of Five (zero violations) and Bioavailability score ≥0.55. Next, target prediction was performed for the selected *C. terminalis* constituents. Potential target proteins were predicted using SwissTargetPrediction, with *Homo sapiens* specified as the target species and a probability cutoff ≥0.05, and PharmMapper, using the top 300 ranked targets based on the Normalized Fit Score. Duplicate targets were removed, and the targets obtained from both databases were combined and verified using UniProt.

For pain and inflammation, potential disease-related genes were retrieved from DisGeNET, GeneCards, and OMIM using the predefined search terms “pain” and “inflammation.” The collected targets were then merged, duplicates were removed, and all targets were verified using UniProt.

*Cordyline terminalis* interrelated targets and pain and inflammation associated targets were intersected using a Venn diagram to recognize the overlapping gene set. These shared genes were assumed to be the core targets potentially linking *C. terminalis* to pain and inflammation signaling pathways. The overlapping genes were submitted to the STRING database for protein-protein interaction networks construction and functional enrichment investigation using the highest confidence interaction score of 0.9 as the minimum required threshold.

Network visualization and analysis were performed using Cytoscape 3.10.2, with degree centrality applied as the main criterion to identify hub genes with greater interaction intensity. The same overlapping gene set was then uploaded to Metascape for enrichment analysis, including Gene Ontology (GO) biological process analysis and Kyoto Encyclopedia of Genes and Genomes (KEGG) pathway enrichment.

### 2.5 Statistic analysis

The experiment was arranged in a completely randomized design with 10 replicates for each treatment. Data were examined for normal distribution using the Shapiro–Wilk test (Shapiro and Wilk, 1965), while homogeneity of variances was evaluated by Levene’s test (Hardin, 1995). Statistical differences among treatments were determined by one-way analysis of variance (ANOVA), followed by Tukey’s *post hoc* test (Chmiel et al., 2022) at a significance level of p < 0.05. All statistical analyses were conducted using GraphPad Prism version 8 (GraphPad Software, San Diego, CA, United States).

## 3 Results

The analysis of palm waste biochar (Table 1) shows that it is rich in important nutrients and organic materials that can support plant growth. It contains a high level of carbon (46%) and organic matter (50.47%)**,** which helps improve soil structure, increase water holding capacity, and encourage beneficial soil microbes. It also provides essential nutrients such as nitrogen (3.18%)**,** phosphorus (2.49%)**,** and potassium (4.70%)**,** making it a good source of slow-release fertilizers. With a C:N ratio of 24.1, the biochar is stable and releases nutrients gradually over time. Its slightly alkaline pH (7.26–7.47) can help reduce soil acidity and create a better environment for root development. Overall, these properties make palm waste biochar a valuable soil amendment that can enhance plant growth and improve soil health.

### 3.1 Effects of BAP (mg/L), biochar (g/L) and their combination on multiple growth parameters of *C. terminalis*

**FIGURE 1:**
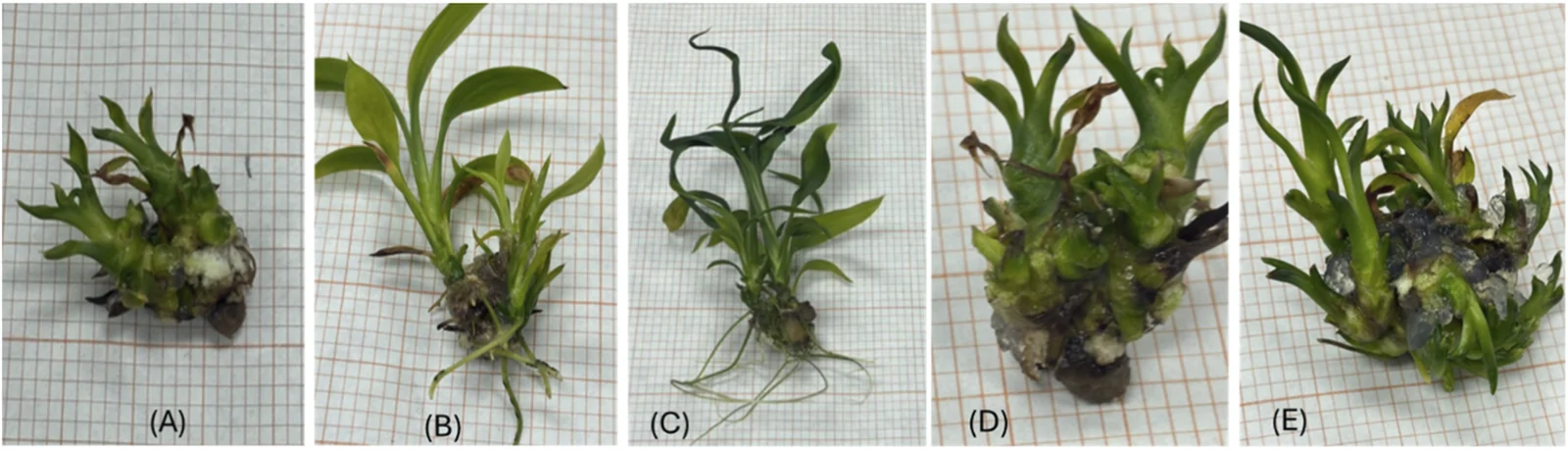
*In vitro* culture of C. terminalis showing multiple shoot formation from shoot tip explants under different treatments. **(A)** Medium supplemented with 1 mg/L 6-benzylaminopurine (BAP); **(B)** 4 g/L biochar; **(C)** 6 g/L biochar; **(D)** 1 mg/L BAP +4 g/L biochar; and **(E)** 1 mg/L BAP +6 g/L biochar.

**FIGURE 2:**
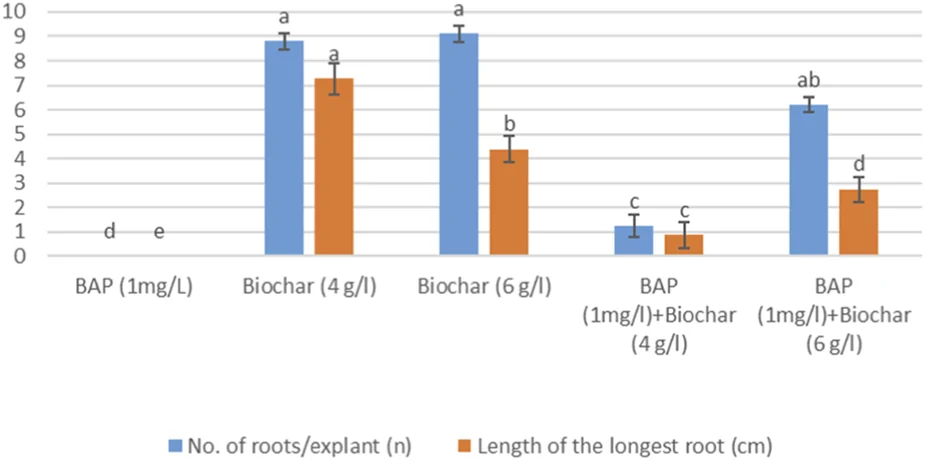
Effect of different concentrations of BAP and Biochar on root number/explant and length of the longest root (cm) induction of *C. terminalis*.

Data presented in Table 2; Figure 2 demonstrated that both BAP and biochar treatments significantly affected shoot proliferation, shoot growth, leaf formation, callus induction, root number, and root length of *C. terminalis* explants. The combination of 1 mg/L BAP with 4 g/L biochar significantly increased the number of shoots per explant (12.1 shoots), explant fresh weight (3.34 g), and number of leaves per explant (47 leaves) compared with all other treatments, including the control medium containing BAP alone without biochar. This treatment also significantly reduced callus formation (18.78%) relative to the control treatment (50%) (Figure 1D). The control medium supplemented with BAP alone produced 9 shoots per explant and 19.6 leaves per explant; however, these values were significantly lower than those obtained with the addition of biochar (Figure 1A). Moreover, the control treatment showed the highest callus percentage, indicating that biochar supplementation improved organogenesis and reduced excessive callus formation. Biochar applied alone significantly promoted shoot elongation compared with all other treatments. The medium containing 4 g/L biochar alone produced the longest shoots (10.82 cm), followed by 6 g/L biochar (8.44 cm), both of which were significantly higher than the control medium and the BAP containing treatments (Figures 1B,C). However, these treatments produced significantly fewer shoots and lower fresh weight values than the combined BAP + biochar treatments. In addition, biochar alone significantly induced root formation without the addition of any auxin (Figures 1B,C). Explants cultured on medium supplemented with 4 g/L biochar produced 8.8 roots per explant with the longest root length reaching 7.26 cm, while 6 g/L biochar produced 9.1 roots per explant with a root length of 4.38 cm (Figures 1B,C, 2). In contrast, the control medium containing BAP alone failed to induce rooting (Figure 1A), recording no root formation. The combination of BAP with biochar reduced rooting efficiency, where 1 mg/L BAP +4 g/L biochar produced only 1.25 roots per explant with a root length of 0.875 cm, whereas 1 mg/L BAP +6 g/L biochar resulted in 6.2 roots per explant with a root length of 2.75 cm (Figures 1D,E, 2). These findings indicate that biochar alone was more effective in stimulating roots than its combination with BAP. Furthermore, biochar only treatments showed no or very low callus formation compared with media containing BAP, suggesting healthier and more organized tissue development.

**TABLE 2:** Effects of biochar (g/L), BAP (mg\L) and their combination on multiple shoot induction of *C. terminalis*.

BAP (mg/L)	Biochar (g/L)	No. Of shoots/explant (n)	Explant’s fresh weight (g.)	Length of the longest shoot (cm)	No. Of leave/explant (n)	Callus (%)
1	0	9 b*	3.21 b	4.04 c	19.6 d	50 a
0	4	4.2 c	2.11. D	10.82 a	24.8 c	0 d
0	6	3.8 c	1.81 e	8.44 ab	27.2 b	0 c
1	4	12.1 a	3.34 a	5.65 bc	47 a	18.78 b
1	6	9.2 b	2.56 c	5.18 bc	33.4 b	10 c

* Means followed by the same letter within a column are not significantly different at p < 0.05 according to Tukey’s *post hoc* test.

### 3.2 Effects of biochar, BAP, and their interaction on photosynthetic pigments

Data in Table 3 showed that, the application of biochar and BAP caused significant differences in chlorophyll and carotenoid contents. BAP alone (1 mg/L) showed significantly the lowest values in all pigments (Chl a, Chl b, total chlorophyll, and carotenoids). Using biochar at 4 g/L significantly increased pigments, and it recorded the highest carotenoid content, which differed significantly from all other treatments. Biochar at 6 g/L alone produced significantly the highest chlorophyll a, while total chlorophyll also remained high. The combination of 1 mg/L BAP +6 g/L biochar showed a significant increase in total chlorophyll and chlorophyll b, representing the highest values in these traits. In contrast, BAP +4 g/L biochar resulted in moderate pigment levels, which were significantly lower than the 6 g/L combination in total chlorophyll and carotenoids.

**TABLE 3:** Effects of biochar (g/L), BAP (mg\L) and their combination on the compositions (mg/100 g fresh weight of leaves) of chlorophyll a (Chl a), chlorophyll b (Chl b), Chl a+Chl b and carotenoids in *C. terminalis* **.**

BAP (mg/L)	Biochar (g/L)	Chl a (mg/100 g F.W.)	Chl b (mg/100 g F.W.)	Chl a+Chl b (mg/100 g F.W.)	Carotenoids (mg/100 g F.W.)
1	0	10.259 e*	10.89 c	21.149 e	13.6 e
0	4	18.37 d	23.43 b	41.8 d	48.69 a
0	6	26.19 a	24.04 b	50.23 b	33.58 b
1	4	23.03 b	24.68 b	47.71 c	21.03 d
1	6	22.43 c	31.92 a	54.35 a	26.44 c

* Means followed by the same letter within a column are not significantly different at p < 0.05 according to Tukey’s *post hoc* test.

### 3.3 Influence of biochar (g/L), BAP (mg/L), and their combined treatment on the composition of *C. terminalis* methanolic extract

GC-MS analysis putatively identified compounds in *C. terminalis* explants cultured under different BAP and biochar treatments (Table 4; Supplementary Tables S1, 2). β-D-glucosyloxyazoxymethane (Cycasin) was the predominant putatively identified compound in all treatments, with the highest levels recorded in biochar-alone treatments at 6 g/L (91.29%) and 4 g/L (89.81%), followed by combined treatments of biochar with BAP at 4 g/L (80.40%) and 6 g/L (77.43%), while the lowest value was observed in the BAP-only treatment (74.13%), Table 4. In contrast, pentadecanoic acid showed a marked treatment-dependent increase, reaching its maximum in the combined treatment of biochar (6 g/L) with BAP (1 mg/L) (23.47%), whereas lower levels were detected in all other treatments. Similarly, 2,3-dihydro-3,5-dihydroxy-6-methyl- 4H-pyran-4-one, was enhanced under the same combined treatment (21.22%), compared with reduced levels in other treatments. Sucrose and 3,4-altrosan were also elevated in the biochar (6 g/L) + BAP treatment (2.95% and 5.84%, respectively), while ethanol, 2-(9-octadecenyloxy)-, (Z)- was detected only in the combined treatments, with the highest abundance in biochar (6 g/L) + BAP (4.58%). Steroidal derivatives, including cholest-7-en-3-one, 4,4-dimethyl-(5α-) (5.00%) and cholestan-3-one, 4,4-dimethyl-(5α-) (4.40%), were exclusively detected in the biochar (4 g/L) + BAP treatment. Additionally, butoxyacetic acid was specific to this treatment (2.19), Table 4. Thymine and 1-heptanol, 2,4-dimethyl-, (R,R)-(+), were uniquely identified in the biochar (6 g/L) + BAP treatment, whereas 3,7,11,15-Tetramethyl-2-hexadecen-1-ol (phytol) (was present only in the BAP treatment (0.86%), Supplementary Table S1. Overall, biochar supplementation, alone or in combination with BAP, significantly altered the metabolic profile of *C. terminalis* explants and promoted the accumulation or induction of several putatively identified metabolites with reported biological relevance.

**TABLE 4:** Effects of biochar (g/L), BAP (mg\L) and their combination on the compositions of methanolic extract prepared from *C. terminalis*.

Compound name	Area, %				
	BAP (1 mg/L)	Biochar (4 g/L)	Biochar (6 g/L)	Biochar (4 g/L + BAP 1 mg/L)	Biochar (6 g/L + BAP 1 mg/L)
β -D-Glucosyloxyazoxymethane	74.13e	89.81b	91.29 a	80.40c	77.43 d
Pentadecanoic acid	3.59c	1.76d	2.00 d	5.73b	23.47a
4H-pyran-4-one, 2,3-dihydro-3,5-dihydroxy-6-methyl-	1.22d	2.06b	1.57c	–	21.22 a
Sucrose	2.23b	–	0.62b	–	2.95a
3,4-Altrosan	–	0.96b	0.97b	–	5.84a
Ethanol, 2-(9-octadecenyloxy)-, (Z)-	–	–	–	2.28b	4.58a
Cholest-7-en-3-one, 4,4-dimethyl-, (5α-)	–	–	–	5.00 a	–
Cholestan-3-one, 4,4-dimethyl-, (5α-)	–	–	–	4.40 a	–
Thymine	–	–	–	–	3.60a
n-Propyl nonyl ether	–	0.22b	0.21b	–	2.39a
1-Heptanol, 2,4-dimethyl-, (R,R)-(+)	–	–	–	–	2.54 a
Butoxyacetic acid	–	–	–	2.19a	–

* Means followed by the same letter within a column are not significantly different at p < 0.05 according to Turkey’s *post hoc* test.

### 3.4 Network pharmacology

**FIGURE 3:**
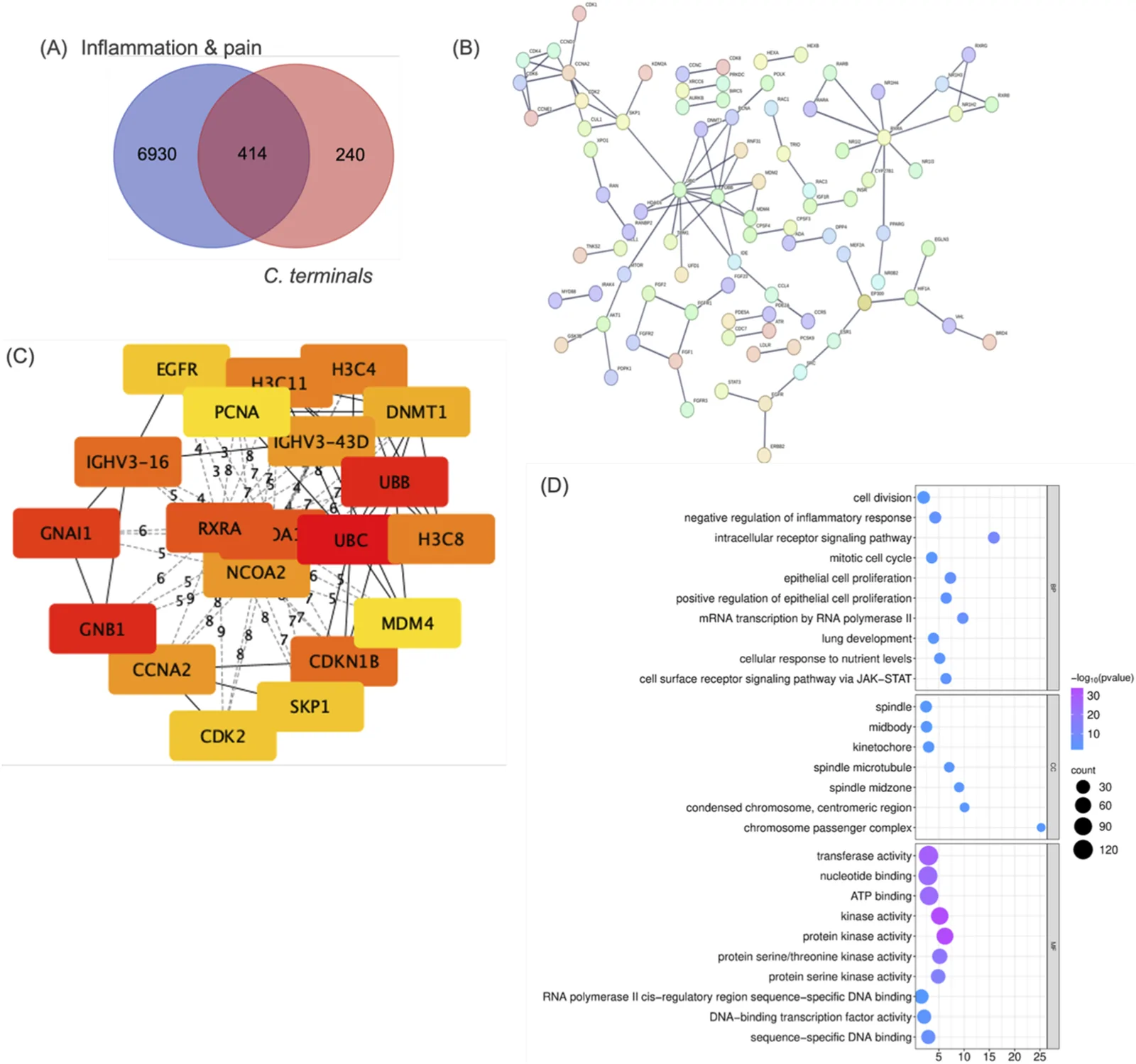
**(A)** Intersection analysis between *C. terminalis* targets and pain and inflammation associated genes using VEN DIAG, **(B)** STRING investigation, **(C)** Network Construction by Cytoscape recognized the top genes and **(D)** Gene Ontology enrichment analysis of the common targets divided into three categories: BP, CC, and MF.

Swiss Target Prediction revealed 463 hits while Pharm mapper revealed 1899 unique hits. Merging both and removing duplicated revealed 2384 unique values remain for verification. From these, 255 IDs not mapped while 2,129 were mapped from which only 654 genes were human mapped. Gene cards showed 17,900 results for Pain, 17,010 results for inflammation. Finally, collection 23,436 unique genes remained. OMIM revealed 798 entries for Pain, 1,188 entries for inflammation. After collection and removing duplicates 365 unique genes remained. While DisGeNET showed 42 unique values remain. After collection from all data bases and removing duplicate, UniProt 14,777 IDs were not mapped while mapped 7,368 IDs from which 7,344 related to human. The overlapping area in Venn diagram included 414 common targets. These are the shared targets between *C. terminalis* and inflammation/pain. Therefore, these 414 genes are considered the potential therapeutic targets through which *C. terminalis* may exert anti-inflammatory and analgesic effects (Figure 3A). STRING investigation revealed Number of nodes was 412, number of edges 90, average node degree:0.437, avg. Local clustering coefficient:0.14, expected number of edges: 21, PPI enrichment p-value: <1.0 ^e−16^. Figure 3B shows the protein–protein interaction network constructed from the 414 overlapping targets identified in Venn suggesting that the action of *C. terminalis* against inflammation/pain is likely multi-target and multi-pathway, rather than being mediated by a single protein. Figure 3C is the hub targets or core targets. The color intensity indicates their relative importance in the network. Targets such as UBC (Ubiquitin C) and UBB (Ubiquitin B) suggest that ubiquitin-related regulation may be important in the biological effects of *C. terminalis*. Other targets, such as EGFR, PCNA, CDK2, CCNA2, and CDKN1B, are associated with cell proliferation, cell cycle control, and tissue repair processes. Figure 3D presents the Gene Ontology enrichment analysis of the common targets allocated into three categories: Biological processes (BP), Cellular Component (CC), and Molecular Function (MF). BP enriched among the common targets included Cell division and mitotic cell cycle, suggesting that the targets may influence cell proliferation and tissue repair. Negative regulation of inflammatory response, which directly supports the possible anti-inflammatory activity of *C. terminalis*. Cell surface receptor signaling pathway via JAK–STAT, suggesting involvement of inflammatory and immune signaling pathways. As for the Molecular Function (MF), important enriched functions included transferase activity, nucleotide binding, ATP binding, kinase activity, protein kinase activity, protein serine/threonine kinase activity, and DNA-binding transcription factor activity. These functions suggest that many targets are enzymes or signaling proteins. Kinases are especially important because they regulate inflammatory pathways, pain signaling, cell survival, and immune responses. The size of each dot reflects the number of genes involved in that term, while the color reflects the statistical significance, shown as −log10 (p-value). Larger and more intensely colored dots indicate more important enriched functions. Gene Ontology enrichment analysis suggests that *C. terminalis* may exert anti-inflammatory and analgesic effects through multiple molecular targets. The most important shared targets are involved in inflammatory regulation, JAK–STAT signaling, kinase activity, cell cycle control, epithelial proliferation, and receptor-mediated signaling.

**FIGURE 4:**
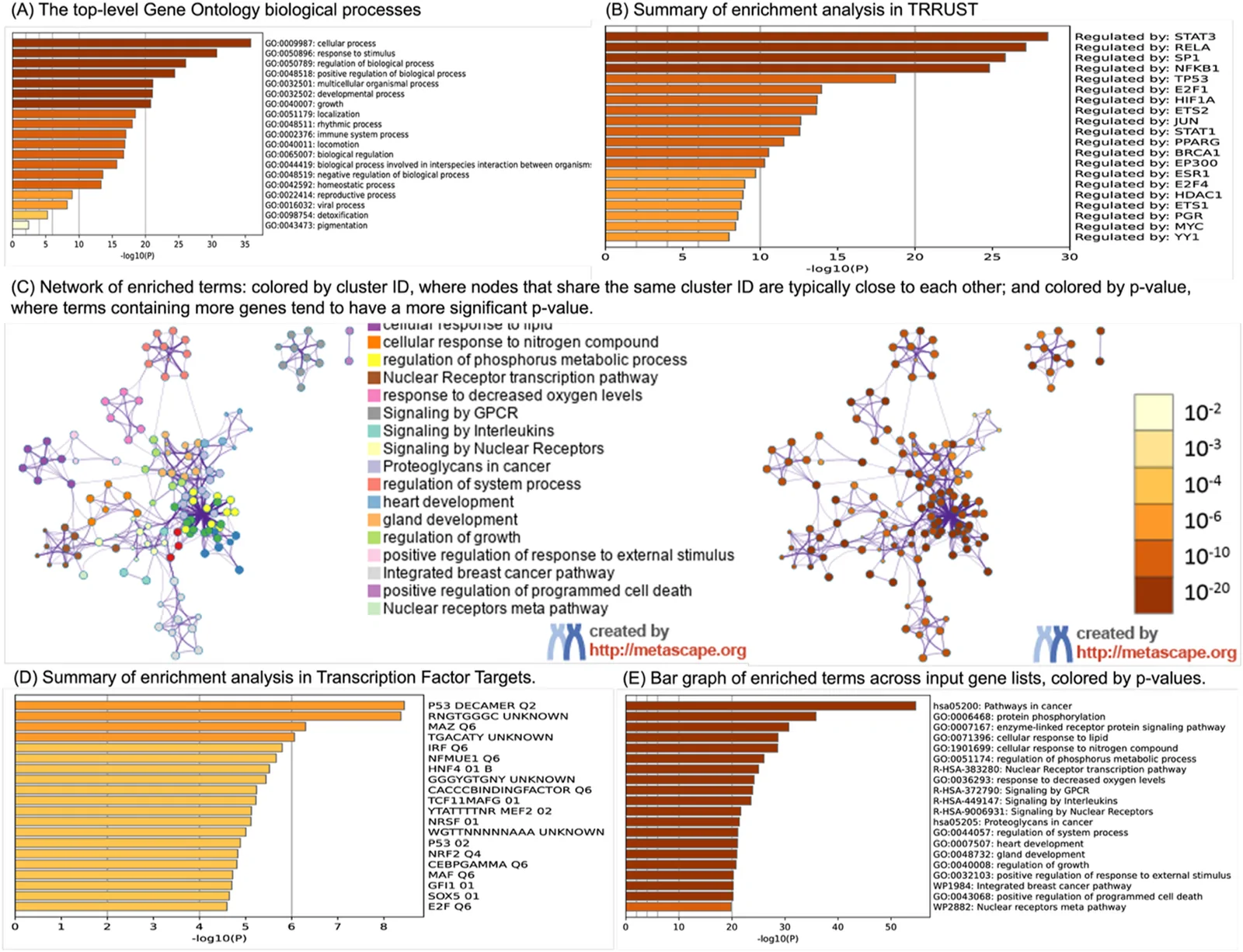
**(A)** Gene Ontology biological processes, **(B)** Enrichment analysis using the TRRUST database, **(C)** Enriched terms associated with the common genes, color-coded by cluster ID and p-value, **(D)** Enrichment analysis in transcription factor targets and **(E)** enriched pathways and biological terms color by p-value.

Similarly, Metascape enrichment analysis of the common genes. It explains the major biological processes, transcriptional regulators, enriched pathways, and functional gene clusters that may be involved in the activity of the studied plant/compound against the investigated condition. Figure 4A shows the most significant top-level Gene Ontology biological processes enriched among the input genes. The x-axis represents −log10(P-value). A higher value means stronger statistical significance. Therefore, the longer the bar, the more significantly that biological process is enriched. The most enriched biological processes include Cellular process, Response to stimulus, Regulation of biological process and positive regulation of biological process.

Figure 4B shows enrichment analysis using the TRRUST database, which identifies transcription factors that may regulate the input genes. The top enriched regulators included STAT3 which is one of the most important transcription factors in inflammation, immunity, cell survival, and tissue repair. Another top enriched regulator is RELA which is a major component of the NF-κB pathway. Other included SP1, NFKB1, TP53 among others.

Figure 4C shows a network map of enriched biological terms generated by Metascape in which Each node represents an enriched term or pathway. The left network is colored by cluster ID. Important clusters include Cellular response to lipid, Cellular response to nitrogen compound, Regulation of phosphorus metabolic process and nuclear receptor transcription pathway and signaling by nuclear receptors Also, the right network is colored by P-value. Darker colors indicate more statistically significant enrichment. Therefore, clusters with darker nodes are likely to represent the strongest biological themes in the gene set.

Figure 4D shows enrichment of transcription factor target motifs among the input genes. The x-axis again represents −log10(P-value), so longer bars indicate more significant enrichment. Important enriched transcription factor motifs included p53-regulated genes, suggesting involvement of stress response, apoptosis, DNA damage response, and cell cycle regulation. Other transcription factor target included MAZ, IRF, NFY family motifs, STAT-related motif, NRF2. The presence of NRF2-related enrichment is particularly important because NRF2 is a major antioxidant transcription factor that regulates cytoprotective genes.

Figure 4E summarizes the most significantly enriched pathways and biological terms across the input gene list. The most enriched term is Pathways in cancer. Other enriched pathways include Protein phosphorylation, Enzyme-linked receptor protein signaling pathway, Cellular response to lipid.

## 4 Discussion

The chemical composition of palm waste biochar indicates its strong potential as a beneficial additive in plant tissue culture. Its high carbon and organic matter content helps improve the physical and biological properties of the culture medium, potentially enhancing aeration and moisture retention around the explants. The presence of essential macronutrients such as nitrogen, phosphorus, and potassium also provides a slow and steady supply of nutrients, which is particularly valuable during prolonged *in vitro* cultivation. Similar findings were noted that biochar can act as both a structural and nutritional enhancer in plant culture systems (Manolikaki and Diamadopoulos, 2019; Gul-Lalay et al., 2024; Ali et al., 2025; Rathinapriya et al., 2025).

In this study, the addition of biochar significantly improved the multiplication performance of *C. terminalis*. The combination of 1 mg/L BAP and 4 g/L biochar resulted in the highest number of shoots, leaf production, and fresh biomass, demonstrating a synergistic effect between the cytokinin and biochar. Biochar likely enhanced nutrient availability and moderated the hormonal balance in the medium, making it more favorable for organogenesis. This agrees with the work of (Murtaza et al., 2024; Çığ et al., 2021; Wiszniewska et al., 2023b), whos suggested that biochar can adsorb excess hormones, reducing their inhibitory effects while gradually releasing them to maintain equilibrium.

Interestingly, explants grown in media containing biochar without BAP showed significantly greater shoot elongation and root development. This suggests that biochar can substitute partially for plant growth regulators by enhancing physiological vigor and reducing stress, especially during early rooting. Similar effects of biochar on root induction were reported (Feng et al., 2021; Sharma et al., 2015; Laura et al., 2023; Niu et al., 2024), where biochar promoted stronger root systems and improved acclimatization success.

The enhancement of photosynthetic pigments by biochar treatments further supports its physiological benefits. Treatments with 4 g/L and 6 g/L biochar showed significantly higher chlorophyll and carotenoid contents compared to BAP alone, indicating greater photosynthetic capacity and stress tolerance. Biochar may improve nutrient uptake, particularly magnesium and nitrogen, which are vital for chlorophyll synthesis (Hossain et al., 2020; Jiang et al., 2022; Chew et al., 2022; Beyyavaş, 2025). Biochar-enriched media encourage better chlorophyll development due to improved cation exchange and nutrient retention.

Secondary metabolite production was also influenced by biochar. GC-MS analysis showed that biochar, especially when combined with BAP, led to the accumulation of a wider range of bioactive compounds in methanolic extract. The combination of 6 g/L biochar and 1 mg/L BAP produced the richest chemical profile, including glucosides, fatty acids, and sterol-related compounds. Biochar’s porous structure may act as a micro-reservoir, enhancing metabolic activity by stabilizing hormone distribution and nutrient availability. Biochar can stimulate secondary metabolism by inducing mild physiological stress and improving nutrient dynamics (Alphianti et al., 2025b; Saleem et al., 2023). A comparative analysis with the phytochemical composition of wild *C. terminalis* indicates substantial modulation of secondary metabolism under *in vitro* conditions. Wild specimens have been reported to predominantly accumulate steroidal saponins, flavonoids, phenolic acids, tannins, and phytosterols, which are generally associated with strong antioxidant and cytoprotective functions (Tematio Fouedjou et al., 2023a; Hossain and Nagooru, 2011). In contrast, the present study demonstrates a shift toward increased representation of oxygenated fatty acids, sterol derivatives, and glucoside type metabolites under biochar and cytokinin mediated culture conditions. This deviation suggests that *in vitro* manipulation of growth conditions can reprogram secondary metabolic pathways, leading to an altered metabolite distribution relative to field grown plants (Baky et al., 2026; Dhar et al., 2015).

Furthermore, when compared with previously reported plant tissue culture and elicitation systems in *C. terminalis* and phylogenetically related monocot species, biochar supplementation appears to exert a broader metabolic regulatory effect (Wang et al., 2025; Yang et al., 2025). Conventional elicitation approaches, including the application of plant growth regulators (e.g., BAP, kinetin, and NAA), have primarily been associated with enhanced biomass production and moderate increases in phenolic and flavonoid biosynthesis via activation of the phenylpropanoid pathway (Li et al., 2025; Jamwal et al., 2018). The present findings suggest that biochar, particularly in combination with BAP, functions as a multi modal elicitor capable of simultaneously influencing carbohydrate conjugation, lipid metabolism, and sterol biosynthesis, resulting in a more complex and diversified secondary metabolite profile.

The anti-inflammatory and analgesic activities exhibited by the extract may be associated with several bioactive constituents identified through GC-MS analysis. Notably, phytol was identified in the BAP treated cultures has been widely reported to possess potent anti-inflammatory and antinociceptive activities (Is et al., 2020) Its pharmacological effects are inhibits the inflammatory response by reducing cytokine production and oxidative stress (Silva et al., 2014).

Furthermore, the steroidal compounds cholest-7-en-3-one, 4,4-dimethyl-(5α-) and cholestan-3-one, 4,4-dimethyl-(5α-) were detected exclusively in the biochar (4 g L^-1^) + BAP (1 mg L^-1^) treatment. These steroidal derivatives are known to interfere with inflammatory signaling cascades and inhibit the production of inflammatory mediators, suggesting their possible contribution to the enhanced anti-inflammatory potential of this treatment (Dembitsky and Terent’ev, 2026). In addition, pentadecanoic acid was detected in all treatments and accumulated at its highest level in the biochar (6 g L^-1^) + BAP (1 mg L^-1^) treatment. This odd-chain fatty acid has been reported to exhibit inflammation regulating properties and is associated with reduced levels of inflammatory biomarkers (Venn-Watson and Schork, 2023). The relatively high abundance of pentadecanoic acid, together with the occurrence of phytol and steroidal constituents, suggests that these compounds may act synergistically to enhance the anti-inflammatory and analgesic activities of the extracts. Therefore, the observed biological activities could be attributed to the presence and combined effects of phytol, steroidal compounds, and pentadecanoic acid, which are well documented for their anti-inflammatory and antinociceptive properties (Li et al., 2023).

The predominance of β-D-Glucosyloxyazoxymethane, commonly known as cycasin, in the methanol leaf extract may be associated with its polar glycosidic character, which could favor extraction in methanol. Cycasin is a known naturally occurring azoxyglycoside, mainly reported from cycad plants, where it is considered part of the plant chemical defense system (Castillo-Guevara and Rico-Gray, 2003). Cycasin is mainly discussed in the literature from a toxicological perspective, since its aglycone methylazoxymethanol is a cycad-derived genotoxin and potent DNA-alkylating agent capable of inducing O6-methylguanine DNA lesions (Verheijen et al., 2024). Since previous phytochemical studies on *Cordyline terminalis* syn *Cordyline fruticosa* have mainly reported steroidal saponins, sapogenins, flavonoids and related phenolic constituents, the detection of cycasin in the present study appears unusual and reported for the first time in this species, however, this finding should be regarded as a putative GC-MS-based identification that warrants further confirmation using targeted LC-MS/MS, authentic reference standard or NMR analysis.

## 5 Conclusion

This study showed that palm waste biochar can play an important role in improving *in vitro* growth of *C. terminalis*. When used with BAP, biochar helped produce more shoots, leaves, and stronger plantlets. Biochar alone also supported better root growth and increased chlorophyll levels, which means the plants were healthier and more active. The combination of biochar and BAP even improved the methanolic extract composition, resulting in richer and more diverse compounds. Overall, biochar proved to be a useful, natural addition to the culture medium, enhancing both growth and metabolic quality. Network pharmacology analysis identified 414 potential targets through which *C. terminalis* may exert anti-inflammatory and analgesic effects. Enrichment analyses revealed that these targets are mainly involved in inflammatory regulation, JAK–STAT signaling, kinase activity, immune response, oxidative stress, apoptosis, hypoxia response, GPCR signaling, interleukin signaling, and nuclear receptor pathways. These findings support the traditional use of *Cordyline* species in treating inflammation-related conditions and suggest that *C. terminalis* acts through a multi-target and multi-pathway mechanism.

## 6 Future work

More studies are still needed to understand exactly how biochar works inside the culture medium. Future research should test different types and sizes of biochar to find the best formula for tissue culture. It would also be helpful to study how biochar-treated plantlets perform during acclimatization and after being transferred to soil. Furthermore, exploring how biochar affects genes related to growth and secondary metabolites could open new opportunities for improving medicinal and ornamental plants *in vitro*. Finally, future studies are needed to validate the network pharmacology findings through *in vivo* animal experiments using an appropriate inflammatory and pain model to evaluate the anti-inflammatory and analgesic activities of *C. terminalis* extract. In addition, the expression of key predicted targets and pathways, such as JAK–STAT, NF-κB, GPCR signaling, interleukin signaling, and oxidative stress-related markers, should be investigated which would help to confirm the predicted mechanisms and support the potential therapeutic use of *C. terminalis* as a natural anti-inflammatory and analgesic agent.

## Data Availability

The raw data supporting the conclusions of this article will be made available by the authors, without undue reservation.
