# Supplementary material for: Improvement of Cordyline terminalis micropropagation and bioactive compounds using biochar associated with pain and inflammation relief
Source: Front Pharmacol. 2026 Aug 14;17:1882659. doi: 10.3389/fphar.2026.1882659 (PMC13522177; doi:10.3389/fphar.2026.1882659)
Supplement: Supplementary file 1 [file Table1.docx]

**Supplementary Table 1.** Influence of biochar concentration on the phytochemical profile of methanol extracts from *in vitro C. terminalis* plantlets.

| **Area%** | **Compound name** | **RT** | **BAP mg/L** | **Biochar g/L** |
| --- | --- | --- | --- | --- |
| 1.22 | 4H-Pyran-4-one, 2,3-dihydro-3,5-dihydroxy-6-methyl- | 8.102 | 1 | 0 |
| 2.23 | Sucrose | 12.696 |  |  |
| 1.63 | 1,2,3,4-Cyclopentanetetrol, (1. α.,2.β ,3.β ,4.α)- | 13.317 |  |  |
| 0.86 | 3,7,11,15-Tetramethyl-2-hexadecen-1-ol | 16.445 |  |  |
| 3.59 | Pentadecanoic acid | 17.853 |  |  |
| 74.13 | β-D-Glucosyloxyazoxymethane | 18.395 |  |  |
| 0.45 | β -D-Mannofuranoside, 1-O-(10-undecenyl)- | 19.054 |  |  |
| 0.83 | [1,1'-Bicyclopropyl]-2-octanoic acid, 2'-hexyl-, methyl ester | 19.490 |  |  |
| 0.55 | n-Hexadecanoic acid | 19.735 |  |  |
| 1.33 | 1,3-Dioxolane, 2,2-dimethyl-4-[[(2-methylhexadecyl)oxy]methyl]- | 19.871 |  |  |
| 0.93 | 9-Octadecenoic acid (Z)-, phenylmethyl ester | 22.185 |  |  |
| 1.54 | Cyclononasiloxane, octadecamethyl- | 23.108 |  |  |
| 2.06 | 4H-Pyran-4-one, 2,3-dihydro-3,5-dihydroxy-6-methyl- | 8.095 | 0 | 4 |
| 0.29 | n-Propyl nonyl ether | 10.801 |  |  |
| 0.40 | 2,5-Methylene-d,l-rhamnitol | 12.720 |  |  |
| 0.96 | 3,4-Altrosan | 13.308 |  |  |
| 0.22 | n-Propyl nonyl ether | 15.984 |  |  |
| 0.49 | Heptadecanal | 16.446 |  |  |
| 0.20 | 9,9-Dimethoxybicyclo[3.3.1]nona-2,4-dione | 16.896 |  |  |
| 1.76 | Pentadecanoic acid | 17.854 |  |  |
| 89.81 | β-D-Glucosyloxyazoxymethane | 18.566 |  |  |
| 1.97 | β-D-Mannofuranoside, 1-O-(10-undecenyl)- | 19.492 |  |  |
| 0.22 | 1-Methoxy-3-(2-trimethylsilyloxyethyl)octane | 21.475 |  |  |
| 0.80 | Ergostan-6-one, 3,25-bis(acetyloxy)-5-hydroxy-, (3β,5α)- | 22.982 |  |  |
| 0.50 | β-Sitosterol | 24.640 |  |  |
| 0.32 | Cholest-4-ene, 3β-(methoxymethoxy)- | 29.940 |  |  |
| 1.57 | 4H-Pyran-4-one, 2,3-dihydro-3,5-dihydroxy-6-methyl- | 8.117 | 0 | 6 |
| 0.62 | Sucrose | 12.783 |  |  |
| 0.97 | 3,4-Altrosan | 13.331 |  |  |
| 0.21 | n-Propyl nonyl ether | 15.984 |  |  |
| 0.40 | Z-8-Octadecen-1-ol acetate | 16.445 |  |  |
| 2.00 | Pentadecanoic acid | 17.854 |  |  |
| 91.29 | β-D-Glucosyloxyazoxymethane | 18.492 |  |  |
| 0.43 | β-D-Mannofuranoside, 1-O-(10-undecenyl)- | 19.493 |  |  |
| 0.95 | Ergostan-6-one, 3,25-bis(acetyloxy)-5-hydroxy-, (3β,5α)- | 22.978 |  |  |
| 0.40 | Cholest-4-ene, 3β-(methoxymethoxy)- | 29.926 |  |  |
| 2.19 | Butoxyacetic acid | 12.722 | 1 | 4 |
| 2.28 | Ethanol, 2-(9-octadecenyloxy)-, (Z)- | 16.445 |  |  |
| 5.73 | Pentadecanoic acid | 17.861 |  |  |
| 80.40 | β-D-Glucosyloxyazoxymethane | 18.297 |  |  |
| 5.00 | Cholest-7-en-3-one, 4,4-dimethyl-, (5α-) | 22.976 |  |  |
| 4.40 | Cholestan-3-one, 4,4-dimethyl-, (5α-) | 24.621 |  |  |
| 3.60 | Thymine | 7.441 | 1 | 6 |
| 21.22 | 4H-Pyran-4-one, 2,3-dihydro-3,5-dihydroxy-6-methyl- | 8.102 |  |  |
| 4.06 | 2-Heptenal, 2-methyl- | 9.977 |  |  |
| 2.39 | n-propyl nonyl ether | 10.835 |  |  |
| 2.95 | Sucrose | 12.696 |  |  |
| 0.40 | 2,5-Methylene-d,l-rhamnitol | 12.72 |  |  |
| 5.84 | 3,4-Altrosan | 13.333 |  |  |
| 2.54 | 1-Heptanol, 2,4-dimethyl-, (R,R)-(+)- | 15.984 |  |  |
| 4.58 | Ethanol, 2-(9-octadecenyloxy)-, (Z)- | 16.445 |  |  |
| 0.49 | Heptadecanal | 16.446 |  |  |
| 23.47 | Pentadecanoic acid | 17.854 |  |  |
| 77.43 | β-D-Glucosyloxyazoxymethane | 18.297 |  |  |
| 7.83 | 9,9-Dimethoxybicyclo[3.3.1]nona-2,4-dione | 19.056 |  |  |
| 9.47 | cis-13,16-Docasadienoic acid | 19.489 |  |  |
| 3.62 | Cyclopropaneoctanoic acid, 2-[[2-[(2-ethylcyclopropyl)methyl]cyclopropyl] methyl]-, methyl ester | 19.546 |  |  |
| 2.14 | (2,2,6-Trimethyl-bicyclo[4.1.0]hept-1-yl)-methanol | 21.482 |  |  |
| 3.58 | Cholest-4-ene, 3β -(methoxymethoxy)- | 29.934 |  |  |

**Supplementary Table 2.** Some putatively-identified GC-MS constituents in *C. terminalis* methanol leaf extract, including molecular formulas, chemical classes and base peaks (m/z), assigned based on mass-spectral library matching and available chromatographic verification

| **Molecular formula** | **Base peak (*m/z*)*** | **Compound class** | **Other known names** | **Compound name** |
| --- | --- | --- | --- | --- |
| C_6_H_8_O_4_ | 43 | Phenolic | DDMP, dihydroxy maltol | 4H-Pyran-4-one, 2,3-dihydro-3,5-dihydroxy-6-methyl- |
| C_12_H_22_O_11_ | 57 | Disaccharide | Sucrose | α-D-Glucopyranosyl-(1→2)-β-D-fructofuranoside |
| C_5_H_10_O_4_ | 60 | Cyclitol | - | 1,2,3,4-Cyclopentanetetrol |
| C_20_H_40_O | 57 | Acyclic Diterpenoid alcohol | Phytol | 3,7,11,15-Tetramethyl-2-hexadecen-1-ol |
| C_15_H_30_O_2_ | 73 | Saturated fatty acid | - | Pentadecanoic acid |
| C_8_H_16_N_2_O_7_ | 73 | Azoxy Glycoside | Cycasin | β-D-Glucosyloxyazoxymethane |
| C₁₇H₃₂O₆ | 73 | alkenyl Mannone glycoside | Farnesyl- β -D-Mannofuranoside | β-D-Mannofuranoside, 1-O-(10-undecenyl)- |
| C_21_H_38_O_2_ | 73 | Cyclopropane fatty acid | - | [1,1'-Bicyclopropyl]-2-octanoic acid, 2'-hexyl-, methyl ester |
| C_16_H_32_O_2_ | 43 | Saturated fatty acid | Palmitic acid | n-Hexadecanoic acid |
| C_25_H_40_O_2_ | 91 | Fatty acid ester | benzyl oleate | 9-Octadecenoic acid phenylmethyl ester |
| C_20_H_42_O | 71 | Diterpenoid alcohol | dihydrophytol | 3,7,11,15-Tetramethyl-hexadecanol |
| C₁₂H₂₆O | 55 | Dialkyl ether | 1-propoxynonane | n-Propyl nonyl ether |
| C_7_H_14_O_5_ | 73 | Fatty sugar alcohol | L-Rhamnitol | 2,5-Methylene-D,L-rhamnitol |
| C_6_H_10_O_5_ | 60 | Bicyclic anyhdrosugar | 3,4-Altrosan | 3,4-Anhydrohexopyranose |
| C_17_H_34_O | 57 | Long-chain aldehyde | Hexadecyl Aldehyde | Heptadecanal |
| C_11_H_16_O_4_ | 84 | Bicyclic ketone | - | 9,9-Dimethoxybicyclo[3.3.1] nona-2,4-dione |
| C_32_H_52_O_6_ | 55 | Steroidal compound | - | Ergostan-6-one, 3,25-bis(acetyloxy)-5-hydroxy-, (3β,5 α)- |
| C_29_H_50_O | 43 | Phytosterol | - | β-Sitosterol |
| C₂₉H₅₀O₂ | 430 | Steroidal ether | (3β)-3-(Methoxymethoxy)cholest-4-ene | Cholest-4-ene, 3β-(methoxymethoxy)- |
| C_20_H_38_O_2_ | 57 | Fatty alcohol ester | (8Z)-8-Octadecenyl acetate | Z-8-Octadecen-1-ol acetate |
| C_6_H_12_O_3_ | 57 | Carboxylic acid derivative | - | Butoxyacetic acid |
| C_20_H_40_O_2_ | 73 | Fatty ether alcohol | oleyl alcohol ethoxylate | Ethanol, 2-(9-octadecenyloxy)- (Z)- |
| C_29_H_48_O | 57 | Steroidal ketone | 4,4-dimethyl-5α-cholest-7-en-3-one | Cholest-7-en-3-one, 4,4-dimethyl- |
| C_29_H_50_O | 57 | Steroidal ketone | 4,4-dimethyl-5α-cholestan-3-one) | Cholestan-3-one, 4,4-dimethyl- |
| C_5_H_6_N_2_O_2_ | 126 | Pyrimidine base | 5-methyluracil | Thymine |
| C_8_H_14_O | 97 | Aldehyde | - | 2-Heptenal, 2-methyl- |
| C_9_H_20_O | 84 | Alcohol | - | 1-Heptanol, 2,4-dimethyl- |
| C_20_H_40_O_2_ | 84 | Long chain alcohol | 2-[(Z)-9-Octadecenyloxy]ethanol | Ethanol, 2-(9-octadecenyloxy)- (Z)- |
